# A Case Report of Bilateral Optic Perineuritis With Idiopathic Intracranial Hypertension: Challenges in Diagnosis and Management

**DOI:** 10.7759/cureus.54692

**Published:** 2024-02-22

**Authors:** Nurul-Farah H Shahrudin, Julieana Muhammed, Wan-Hazabbah Wan Hitam, Nur Asma Sapiai, Sanihah Abdul Halim

**Affiliations:** 1 Department of Ophthalmology and Visual Science, School of Medical Sciences, Universiti Sains Malaysia, Kubang Kerian, MYS; 2 Department of Radiology, School of Medical Sciences, Universiti Sains Malaysia, Kubang Kerian, MYS; 3 Department of Brain and Behavior Cluster, School of Medical Sciences, Hospital Universiti Sains Malaysia, Kubang Kerian, MYS

**Keywords:** optic nerve sheath, optic nerve, optic neuritis, idiopathic intracranial hypertension, optic perineuritis

## Abstract

Optic perineuritis (OPN) refers to the inflammation of the optic nerve sheath and it is a rare form of idiopathic orbital inflammatory disease. We report a rare case of bilateral OPN in an obese female teenager with idiopathic intracranial hypertension (IIH). She was initially presented with painless bilateral blurring of vision that was progressively worsening for three weeks duration. Visual acuity of both eyes was hand movement with no relative afferent pupillary defect detected. The confrontation visual field test showed central scotoma. Both anterior segments were unremarkable. Fundoscopy showed a swollen optic disc bilaterally, with extensive flame-shaped hemorrhages surrounding the disc area and dot blot hemorrhages in the posterior pole. A magnetic resonance imaging scan of the brain and orbit revealed the presence of bilateral optic nerve sheath enhancement with empty sella turcica. The patient was diagnosed with bilateral OPN with IIH. She received an initial high dose of systemic corticosteroid followed by a slow tapering dose. She was monitored by the neuromedical team for her IIH. She was followed up for about a year. The final best corrected visual acuity in the right eye was 6/36 and the left eye was 6/60. In conclusion, OPN poses challenges in diagnosis and management. This case emphasizes the importance of considering OPN in the differential diagnosis of optic nerve-related symptoms, as prompt recognition and intervention are crucial for favorable outcomes.

## Introduction

Optic perineuritis (OPN) is the inflammatory disease of the optic nerve sheath, a structure that surrounds the optic nerve. Its most common form, primary OPN, is an isolated and idiopathic condition, whereas secondary OPN is considered to be a part of other systemic conditions such as infectious and inflammatory diseases, including syphilis [1], sarcoidosis [2], Wegener’s granulomatosis [3] and Crohn's disease [4].

OPN is frequently misdiagnosed and treated as optic neuritis (ON) due to its near-similar clinical presentations. It is critical to differentiate OPN from ON due to the treatment and prognostic differences between the two conditions. Unlike ON, which involves inflammation of the optic nerve itself, OPN specifically targets the surrounding optic nerve sheath tissues. Generally, most of the patients will present with either acute loss of vision, eye pain, or both [5].

OPN is diagnosed based on clinical and radiographic findings which is the magnetic resonance imaging (MRI). The hallmark imaging finding in OPN is the contrast enhancement of the optic nerve sheath with sparing of the optic nerve [2]. Treatment of OPN typically requires high doses of corticosteroids. Bilateral OPN cases have been reported previously in the literature; however, in this case, we highlighted the rare occurrence of OPN in IIH patients. There is no definite evidence to suggest the relation between the two pathologies, and whether IIH causes OPN, as the two diagnoses have different underlying pathologic mechanisms. In addition, there were no other similar cases reported or found in the literature. We report a rare case of an idiopathic bilateral OPN in an underlying IIH, with poor visual outcome, for future reference.

## Case presentation

An 18-year-old girl with underlying obesity and IIH presented with a painless blurring of vision in both eyes for three weeks. It was gradually worsening, and the central vision was affected more compared to the periphery. The symptoms were associated with left-sided headache and nausea. She had an initial history of fever, one week before the ocular onset. The fever lasted for four days and it resolved spontaneously. She denied any association with other systemic illnesses at that time. There was no complaint of eye pain, redness, double visions, floaters, or flashes of light. She also denied having any history of numbness or weakness. This patient has no family history of blindness or similar clinical conditions. A few months earlier, she was diagnosed with IIH by the neuromedical team when she presented with a headache and blurring of vision. Before the diagnosis of IIH, the patient had no other medical condition and was not on any medications. Her vision was 6/18 in both eyes. A fundoscopy examination showed the presence of disc swelling in both eyes. CT scan revealed the elevation of both optic discs with slightly thickened optic nerve. However, there was no space occupying the lesion and no optic nerve enhancement. She refused for lumbar puncture procedure and was started on systemic acetazolamide and close monitoring of IIH symptoms.

**Figure 1:**
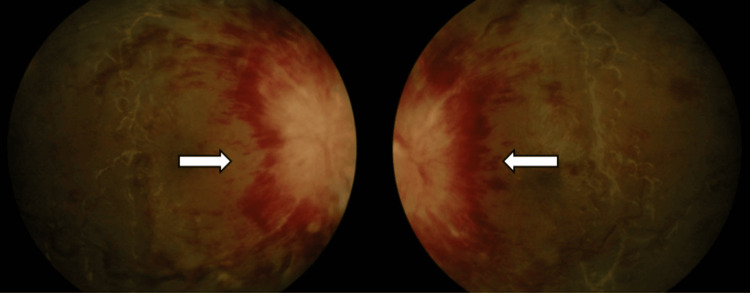
Fundus photo during initial presentation.

On general examination, she was obese with a body mass index of 44.9kg/m^2^. Her vital signs were normal with blood pressure of 130/78mmHg, pulse rate of 83, and random blood sugar level of 5.4 mmol/L. The visual acuity in both eyes was only hand movement. There was no relative afferent pupillary defect detected. Both light brightness and red saturation were reduced in the LE compared to the RE. The confrontation test showed central scotoma with HM vision peripherally in all four quadrants of both eyes. Extraocular muscle movements were normal and there was no proptosis. Both anterior segments were unremarkable. Fundoscopy of both eyes showed an optic disc grossly swollen 360 degrees with obscuration of major vessels. There was the presence of extensive flame-shaped hemorrhage (FSH) surrounding the disc area and of dot blot hemorrhages (DBH) in the posterior pole extending to the mid-peripheral retinal. The macula showed a dull foveal reflex and the vessels appeared tortuous and dilated (Figure 1). There was no retinitis, choroiditis, vasculitis, vitreous, vitreous, or preretinal hemorrhage. Systemic examination was normal.

**Figure 2:**
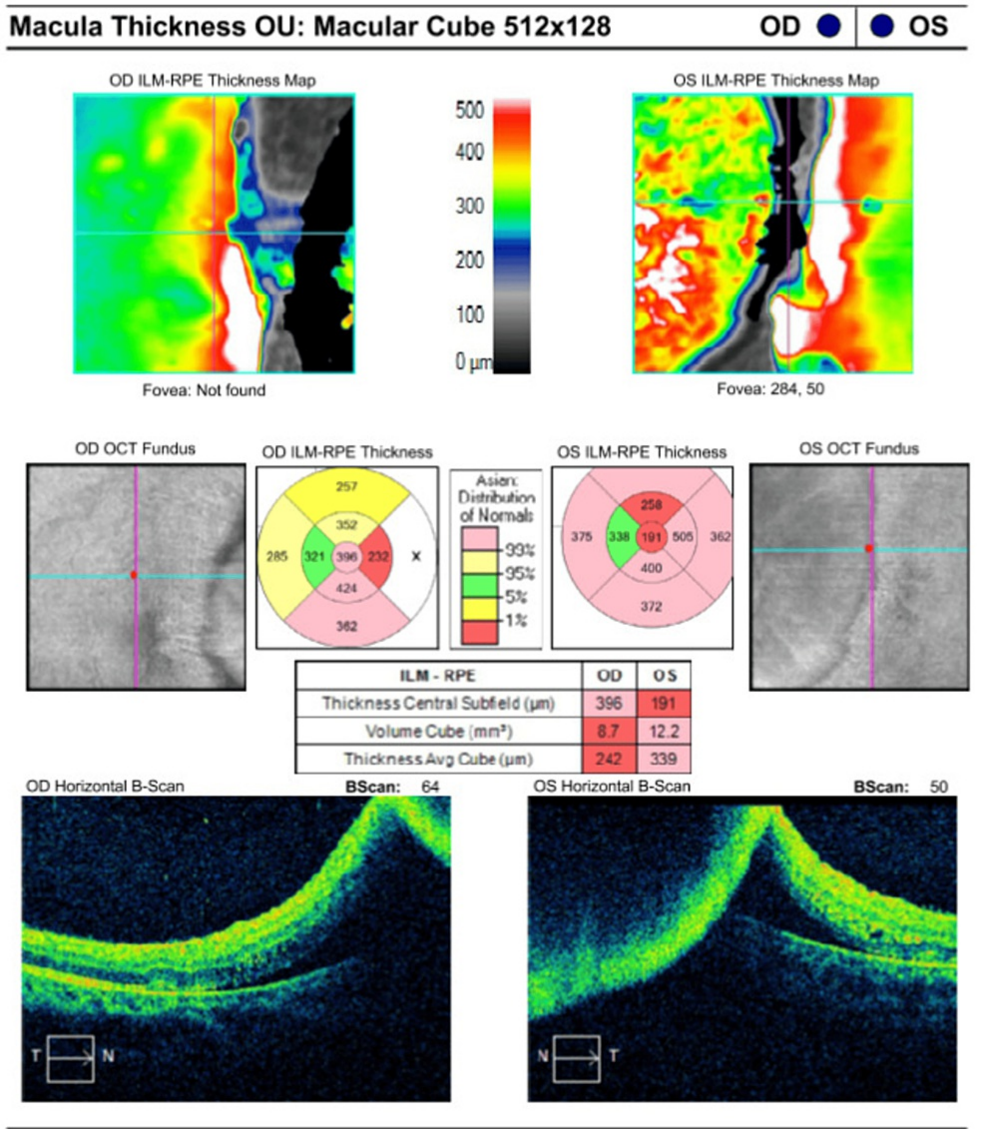
Ocular coherence tomography image of bilateral macula showed the presence of subretinal fluid extending from the optic disc with loss of normal foveal contour.

**Figure 3:**
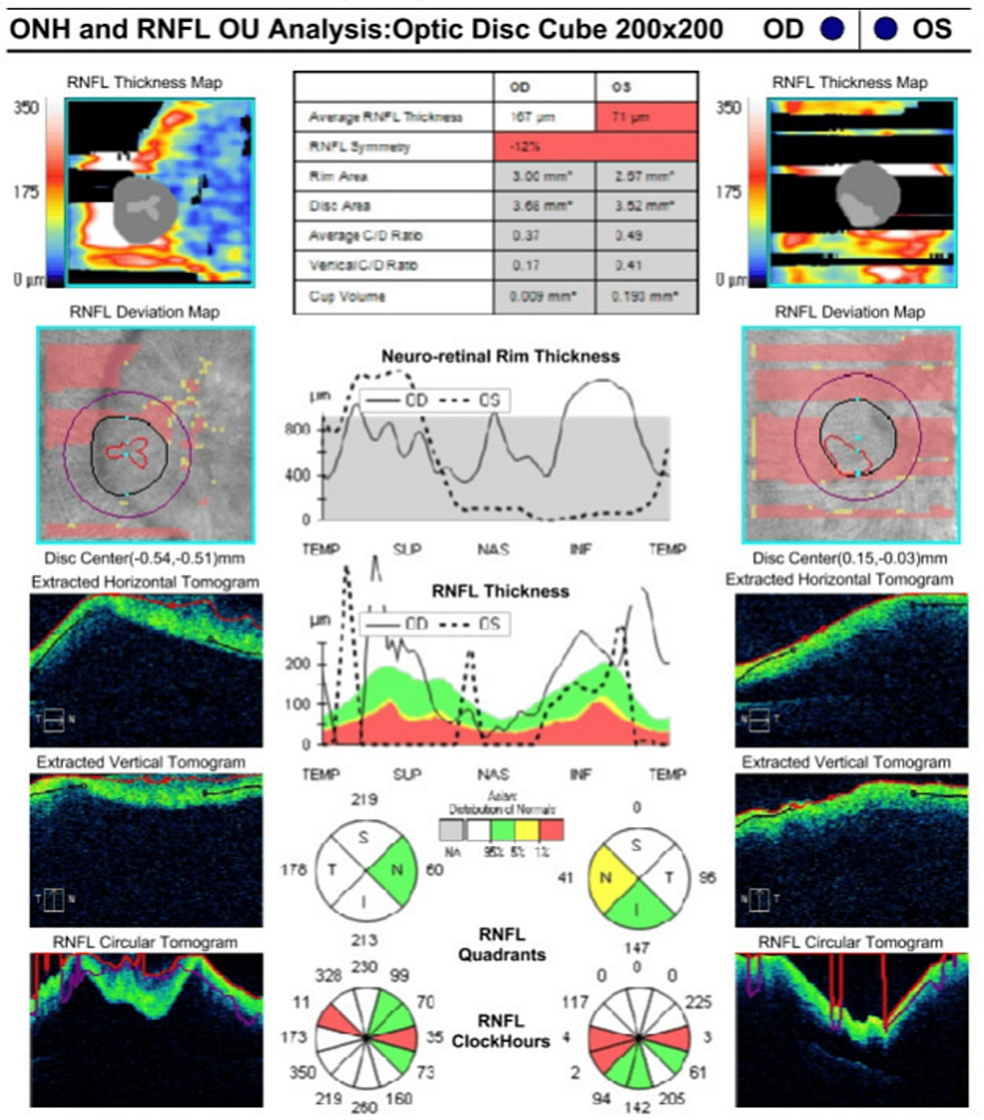
Ocular computed tomography image of optic nerve head and retinal nerve fibre layers showed swollen and abnormal optic disc.

**Figure 4:**
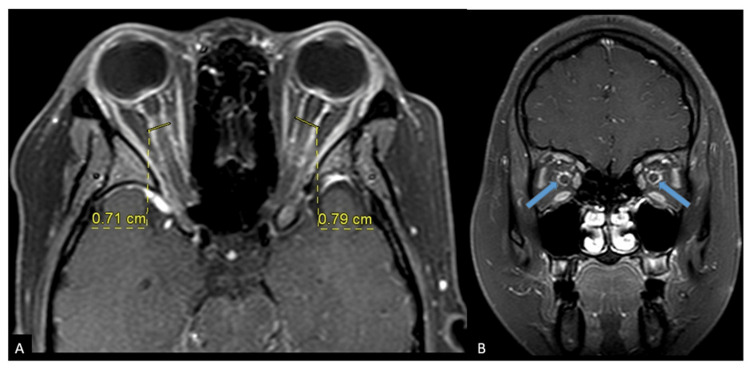
(A) Axial T1 post Gadolinium shows increase bilateral optic nerve sheath diameter. (B) Coronal T1 post Gadolinium shows circumferential thickening of bilateral optic nerve sheath with enhancement.

**Figure 5:**
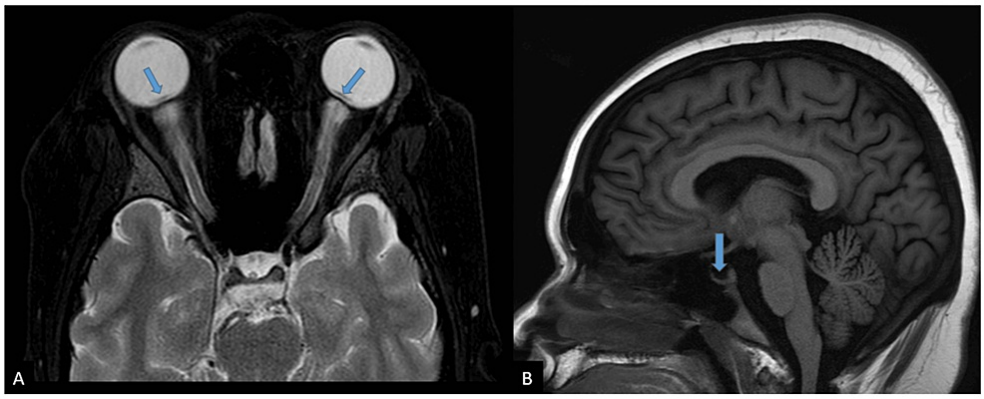
(A) Axial T2 shows flattening of the posterior sclera with intraocular protrusion of the optic nerve head. (B) Sagittal T1 shows empty sella turcica.

Ocular coherence tomography showed bilateral optic disc swelling with the presence of subretinal fluid extending from the disc to the macula area with loss of normal foveal contour (Figures 2, 3). All investigation results are shown in Table 1. MRI showed prominent subarachnoid space around the optic nerves with increased optic nerve sheath diameter, measuring approximately 0.71cm on the right side and 0.79cm on the left side. These were associated with circumferential thickening and enhancement of bilateral optic nerve sheath, which appears as “Tram Tracks” in the axial view and as a “Doughnut” in the coronal view, suggestive of OPN (Figures 4A, 4B). Figure 5A showed bilateral papilloedema with intraocular protrusion of the optic nerve head. There was also evidence of empty sella turcica (Figure 5B) as the patient had an earlier diagnosis of IIH.

**Table 1:** Investigation results

Investigations	Results
Full blood counts	White cell count:14500/microliters (increased)
	Platelet 454000/microliters
	Haemoglobin level of 11.0 gram/deciliters
Liver function test	All parameters were within normal range
Renal profile	All parameters were within normal range
Erythrocyte sedimentation rate	81 millimeters/hour (increased)
C-reactive protein	39 milligrams/decilitre (increased)
Rheumatoid factor	Negative
Anti-nuclear antibody	Negative
Complements C3	1.52 grams/liter (increased)
Complements C3	0.15 grams/liter
Infective screenings (Syphilis, Toxoplasma, Cytomegalovirus, Herpes simplex virus)	Negative
Serum Aquaporin-4 antibody	Negative
Serum Anti-Myelin Oligodendrocyte Glycoprotein antibody	Negative
Mantoux test	0 millimeter (negative)
Chest X-ray	Normal

**Figure 6:**
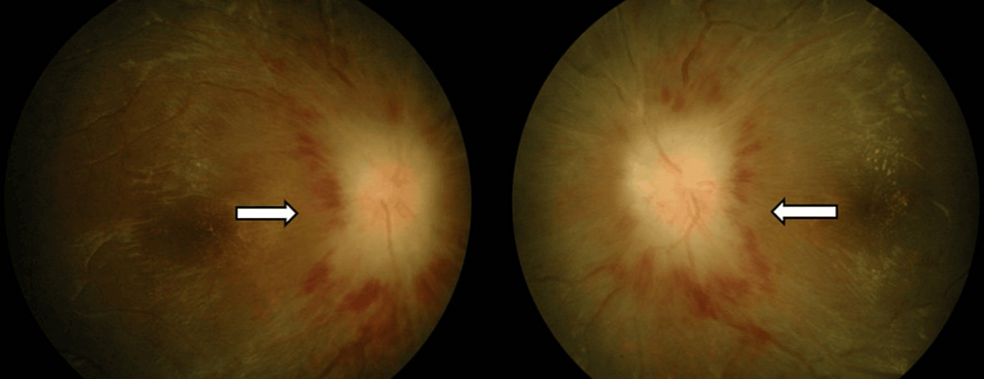
Fundus photo of bilateral eyes at six weeks follow-up following treatment.

**Figure 7:**
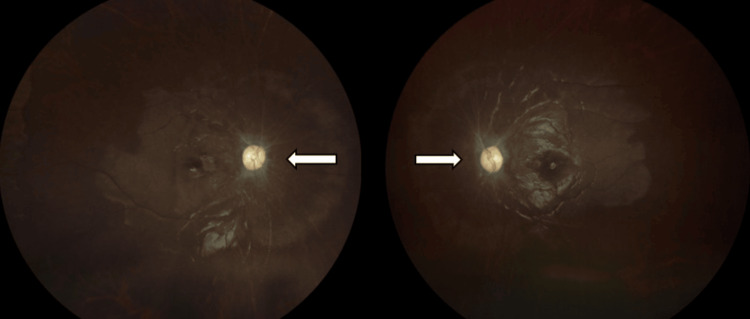
Fundus photo of bilateral eyes after one year follow-up following treatment. The optic disc was pale and atrophic.

This patient was diagnosed with idiopathic bilateral OPN with IIH. She was started with intravenous methylprednisolone 250mg QID for five days duration, followed by oral prednisolone 60mg OD with a slow tapering dose of 10mg weekly for eight weeks. The oral acetazolamide 250mg BD was continued upon her OPN diagnosis with a total duration of six months. She was compliant with the treatment given and there were no unwanted side effects developed from the medications except that she complained of gaining weight about three kilograms throughout her treatment course. At six weeks follow-up, there was only minor improvement of her vision from HM to counting fingers bilaterally. However, there were no more headaches with no new ocular complaints. Her fundus revealed swollen and pale OD bilaterally and lesser FSH surrounding the disc area (Figure 6). Both maculae were flat and dull reflexes with the presence of hard exudates. During her follow-up at one year, the general condition of the patient was stable. Her vision in both eyes improved to 6/36 in the RE and 6/60 in the LE. Both optic discs were pale (Figure 7). There was no history of recurrent or relapse since then as her vision remained the same with no new acute ocular complaint or examination findings.

## Discussion

OPN was first described by Edmunds and Lawford in 1883. They reported a histopathologic specimen of an optic nerve with loosely arranged inflammatory infiltrates surrounding the perimeter [6]. Recently, more literature was available to describe the pathology of OPN in greater detail. Dutton et al., in 1985, found that chronic granulomatous inflammation of the optic nerve sheath is the hallmark of idiopathic OPN [7]. In OPN, the visual loss is attributed to secondary ischaemic infarction of the optic nerve due to circumferential compression by the thickened neural sheath, or to vascular occlusion due to vasculitis [8]. The range of symptoms and exam findings is influenced by the varying degrees and locations of inflammation. OPN is often unilateral, yet there have also been documented bilateral cases in the literature. In a review of 14 cases presented to two different centers, only one case was reported as bilateral [2].

One of the important differential diagnoses of OPN is ON. The key distinguishing features of both diseases are characterized by their clinical and radiological findings. In both conditions, patients may be presented with a sudden onset of visual loss associated with pain that is exacerbated by eye movement. However, the onset of visual loss in ON is more acute, and it occurs within days, as compared to the subacute onset in OPN which typically occurs over weeks. Age group is another clinical factor being observed in OPN, as it commonly affects elderly people. Few studies done before reported that in general, the mean age of patients at the onset of OPN is generally higher than the age at the onset of ON, with the average age being 55 years (range 26-64 years). While the age group in ON is commonly around 20 to 45 years old [9]. A case series from 2001 with 14 idiopathic OPN patients revealed a mean age of 41 years (range 24-60) at presentation [2]. Similar to those in ON, most patients are women and disc edema is usually present in both conditions [5]. In our case, we can see a combination of certain features from both conditions which contributed to the initial diagnosis dilemma. She presented at young age onset like most of the ON patients, with subacute onset of bilateral visual loss. Also, the coexistence of IIH which was diagnosed earlier making the diagnosis even more challenging. Thus, in this case, the neuroimaging findings are very crucial to differentiate it from other condition, and making the final diagnosis of OPN.

IIH may be seen in any gender or age group but has a high predilection for females of childbearing age, especially those with underlying obesity. A modified Dandy criterion was used to diagnose IIH. This patient was diagnosed with IIH a few months earlier with subsequent OPN diagnosis. It is not common for a patient to have double optic nerve-related pathology. There was only one near similar case reported earlier in 2021, which was bilateral ON and IIH coexistence in a patient with a recent COVID-19 infection [10]. However, there was no other case was reported that describes the occurrence of OPN in pre-existing IIH patients.

The diagnosis of OPN is usually confirmed with radiographic findings of MRI. Gadolinium-enhanced, fat-saturated T1 MRI of the orbits is the key to diagnosis of OPN [7]. Contrast enhancement of the optic nerve sheath with optic nerve sparing is a hallmark finding in OPN. In some cases of OPN, the substance of the optic nerve also showed enhancement, presumably due to inflammation of intraneural pial septa as well as optic nerve sheath. This perioptic enhancement in OPN appears as “tram tracks” in the axial view and as a “doughnut” in the coronal view [2]. In our case, the MRI image clearly showed the presence of prominent subarachnoid space around the optic nerves with associated circumferential thickening and enhancement of bilateral optic nerve sheath suggestive of OPN. In addition, although it is not commonly done, an optic nerve biopsy can also confirm the diagnosis of OPN [9].

Systemic corticosteroids are the mainstay of treatment for OPN patients. Corticosteroids causes rapid improvement in signs and symptoms; thus, within hours to a day of starting treatment, patients may experience relief from pain and visual symptoms. However, the high-dose corticosteroid must be commenced with slow tapering dose to avoid recurrence of disease. Patient typically requires high doses of corticosteroids, but relapse following therapy discontinuation is common, although spontaneous resolution has been seen [11]. OPN usually does not resolve without treatment. In OPN, corticosteroid therapy improves final visual outcomes, whereas in ON, intravenous corticosteroid treatment simply accelerates visual recovery and has no effect on final visual outcome. However, in our patient, final visual outcome after the steroid therapy was not satisfactory. This is likely due to several identifiable issues such as late and severe presentations, delaying in corticosteroid commencement, and pre-existing IIH. Complete vision loss may result if the diagnosis is not recognized and treatment is not initiated promptly [12]. Prognosis tends to be poorer in patients who have delayed time from vision loss to onset of steroid initiation, as well as patients with a high frequency bout of illness [5].

In 2016, there was one similar case of bilateral OPN reported in a previously healthy young patient. The case presented with a more acute onset of disease which occur within a week, with visual acuity of the right eye was counting fingers at two feet, and left eye was 6/18. The eye examination showed similar findings of disc swelling. Supported by the MRI findings of OPN, she was started on systemic corticosteroid and completed her treatment for six weeks duration. This case portrayed a success in OPN treatment, as it was reported that patient able to obtain a good final visual outcome of 6/6 for both eyes with no history of relapse [13]. Our case showed almost similar clinical presentations; however, we reported a poor visual outcome, owing to certain factors such as previous underlying IIH and with delayed presentations and thus, treatment commencement.

## Conclusions

OPN is a rare disorder that is clinically challenging to diagnose. A thorough clinical assessment with aid from radiological findings may help to distinguish it from ON. It is mostly a treatable disease when early presentation and detection are achieved, with prompt treatment given appropriately. OPN with IIH is rarely seen and thus, increases the challenges in diagnosis and treatment with favorable outcomes.
